# The Use of Digital Health Tools for Health Promotion Among Women With and Without Chronic Diseases: Insights From the 2017-2020 Health Information National Trends Survey

**DOI:** 10.2196/39520

**Published:** 2022-08-19

**Authors:** Kobi V Ajayi, Elizabeth Wachira, Henry K Onyeaka, Tyra Montour, Samson Olowolaju, Whitney Garney

**Affiliations:** 1 Department of Health & Kinesiology Texas A&M University College Station, TX United States; 2 Department of Health and Human Performance Texas A&M University Commerce, TX United States; 3 Department of Psychiatry Massachusetts General Hospital Boston, MA United States; 4 Department of Psychiatry McLean Hospital Belmont, MA United States; 5 Department of Demography College for Health, Community and Policy University of Texas San Antonio, TX United States

**Keywords:** mHealth, health promotion, chronic disease, women, digital health, USA, United States, patient engagement

## Abstract

**Background:**

In the United States, almost 90% of women are at risk of at least one chronic condition. However, the awareness, management, and monitoring of these conditions are low and present a substantial public health problem. Digital health tools can be leveraged to reduce the alarmingly high rates of chronic condition–related mortality and morbidity in women.

**Objective:**

This study aimed to investigate the 4-year trend of digital health use for health promotion among women with chronic conditions in the United States.

**Methods:**

Data for this study were obtained from the 2017 to 2020 iterations of the Health Information Trends Survey 5. Separate weighted logistic regression models were conducted to test the unadjusted and adjusted association of the study variables and each digital health use. The 95% CI, adjusted odds ratio (aOR), and *P* value (.05) were reported. Analysis was conducted using Stata 17 software.

**Results:**

In total, 8573 women were included in this study. The weighted prevalence of the use of a smartphone or tablet for various activities were as follows: track health goals, 50.3% (95% CI 48.4%-52.2%; 3279/7122); make a health decision, 43.6% (95% CI 41.9%-45.3%; 2998/7101); and discuss with a provider, 40% (95% CI 38.2%-41.8%; 2834/7099). In the preceding 12 months, 33% (95% CI 30.9%-35.2%; 1395/4826) of women used an electronic wearable device, 18.7% (95% CI 17.3%-20.2%; 1532/7653) shared health information, and 35.2% (95% CI 33.2%-37.3%; 2262/6349) sent or received an SMS text message with a health professional. Between 2017 and 2020, the weighted prevalence of having 0, 1, and multiple chronic conditions were 37.4% (2718/8564), 33.4% (2776/8564), and 29.3% (3070/8564), respectively. However, slightly above half (52.2%, 95% CI 0.50%-0.53%; 4756/8564) of US women reported having at least one chronic disease. Women with multiple chronic conditions had higher odds of using their tablet or smartphone to achieve a health-related goal (aOR 1.43, 95% CI 1.16-1.77; *P*=.001) and discuss with their provider (aOR 1.55 95% CI 1.20-2.00; *P*=.001) than those without any chronic conditions. Correspondingly, in the past 12 months, the odds of using an electronic wearable device (aOR 1.40, 95% CI 1.00-1.96; *P*=.04), sharing health information (aOR 1.91, 95% CI 1.46-2.51; *P*<.001), and communicating via SMS text messaging with a provider (aOR 1.31, 95% CI 1.02-1.68; *P*=.03) were significantly higher among women with chronic conditions than those without a chronic condition.

**Conclusions:**

This study suggests that women with chronic conditions accept and integrate digital health tools to manage their care. However, certain subpopulations experience a digital disconnect that may exacerbate existing health inequities. Implications for research and opportunities to leverage and integrate digital health tools to prevent, monitor, manage, and treat chronic conditions in women are discussed.

## Introduction

### Background

Although almost 90% of women in the United States are at risk of at least one chronic condition, the awareness, management, and monitoring of these conditions are low and present a substantial public health problem [1-4]. Women bear a disproportionate burden of chronic diseases, and in 2018 alone, the prevalence of multiple chronic conditions was 28.4% versus 25.9% in men [5,6]. Moreover, some chronic conditions, such as heart disease, are the leading causes of death in women, killing 1 in every 5 women [7]. Digital health tools have the potential to advance and monitor chronic conditions, and they represent a substantial opportunity to prevent and reduce the alarmingly high rates of chronic condition–related mortality and morbidity in women [8-10].

In their review, Adedinsewo and colleagues [11] succinctly outlined how artificial intelligence and digital health tools can be leveraged to improve the screening, monitoring, educating, and managing of chronic conditions among women during the life course. The authors note that digital tools can facilitate smoking cessation and cardiometabolic health in the preconception stage, particularly for those at risk of or with preexisting chronic conditions. Similarly, during pregnancy and among postpartum and menopausal women, digital health tools can be advantageous for the remote digital monitoring of blood pressure, telehealth consultation, or other smartphone-based educational interventions [11]. This finding suggests that integrating and using technology to engage and provide care for women living with chronic conditions is key to achieving the Healthy People 2030 objective: to increase the proportion of adults using health information technology, thereby promoting the health and well-being of women [12].

Despite the prevalence of chronic conditions across the life span, less is known about the adoption of digital health use in women. Previous studies have focused on digital health use during the perinatal period or on pregnancy-related chronic conditions [13-17]. Furthermore, other studies merely compare digital health use between men and women without investigating the distinct patterns of digital health use among women [18-20]. Since it is well established that there are gender and sex differences in the development and outcomes of chronic conditions and digital health use, it is imperative to understand how the adult women population with these conditions use digital health tools to manage their health [2,11]. Understanding women’s digital health use for chronic condition management may encourage the responsiveness of public health programs and interventions to women’s health needs to prevent and control these conditions among this population.

### Objective

Therefore, the objective of this study was to investigate the 4-year trend of digital health use for health promotion among women with noncommunicable chronic conditions in the United States, drawing from nationally representative data. This study also aimed to examine the sociodemographic and health-related factors that influence digital health use for health promotion across subpopulations of women in the United States.

## Methods

### Data Source

Data for this study were obtained from the Health Information Trends Survey (HINTS) [21]. HINTS is a nationally representative sample of noninstitutionalized US adults aged ≥18 years and fielded by the National Cancer Institute. HINTS collects data about respondents’ health communication, digital health use, and sociodemographic characteristics. This study used data from HINTS 5, Cycles 1 (2017), 2 (2018), 3 (2019), and 4 (2020) surveys. Cycles 1 and 2 surveys were stratified by postal address to sample residential addresses randomly, whereas Cycles 3 and 4, in addition to mailed surveys, introduced a web-based option, wherein respondents were randomly grouped into (1) a web-based and paper survey option without an additional bonus and (2) a web-only option with an additional bonus. Per HINTS, there was no statistically significant difference between the 2 groups; rather, providing the bonus and using the web-based option increased the sample representativeness by allowing groups, particularly young adults, who would have been otherwise underrepresented in the mail-only option. Detailed information about the HINTS survey and methodology is reported elsewhere [21]. The total number of surveyed respondents and response rates for each year were as follows: 3285 and 32.3% (Cycle 1); 3504 and 32.9% (Cycle 2); 5438 and 30.3% (Cycle 3); and 3865 and 36.7% (Cycle 4). Unless otherwise stated, all variables analyzed in this study were surveyed across the years.

### Measures

#### Digital Health

Following the strategy of Shan and colleagues [18], we analyzed 6 binary (no/yes) outcome variables to measure digital health use (Multimedia Appendix 1). Although Shan and colleagues [18] measured 9 digital health use variables, we deviated by only measuring digital health use among respondents who responded in the affirmative that they owned either a tablet or smartphone, because our focus is on use versus ownership. As a result, our study included respondents who have used their tablet or smartphone to (1) track health-related goals, (2) make health decisions, and (3) discuss with their provider. Respondents were also asked if, in the past 12 months, they had (4) used an electronic wearable device to track their health-related goals, (5) shared their health information with their health provider with an electronic monitoring device or smartphone, and (6) communicated with their provider via SMS text messaging. Of the 6 digital health measures, the question regarding electronic wearable device use was asked in 2019 and 2020, whereas that regarding the use of SMS text messaging for communicating with the provider was only asked from 2017 to 2019. See Multimedia Appendix 1 for detailed information on the variables.

#### Key Independent Variables

Chronic conditions were measured using 2 questions and were modeled following the approach of Greenberg and colleagues [19]. Respondents answered yes-or-no questions on whether their provider told them they had diabetes or high blood sugar, high blood pressure or hypertension, heart-related conditions, lung disease–related conditions, depression or anxiety, and arthritis or rheumatism. Participants were also asked if they had ever had cancer (no/yes; Multimedia Appendix 1). The number of chronic conditions was totaled and then categorized as 0, 1, and 2 or more chronic conditions. However, in contrast to Greenberg and colleagues [19], we analyzed arthritis or rheumatism separately for all models and did not include them in the totaled chronic conditions, because these were dropped in the 2019 and 2020 HINTS iterations.

#### Covariates

Control variables were included based on theoretical and empirical relevance [19,22]. Sociodemographic variables included in the analyses were age (18-34, 35-49, 50-64, or ≥65 years), marital status (not married vs married), income status (<US $20,000, US $20,000-34,999, US $35,000-49,999, US $50,000-74,999, or ≥US $75,000), race/ethnicity (non-Hispanic White, non-Hispanic Black, Hispanic, non-Hispanic Asian/others, or missing), and education (high school degree or below, some college degree, and college degree or above). Health-related variables included health insurance (no/yes), self-reported health status (fair/poor, good, or excellent), regular provider (no/yes), physical activity (<150 vs >150 minutes per week), and smoking status (never, former, or current).

### Statistical Analysis

All analyses used the recommended analytical strategy by the HINTS analyst and applied 200 replicate jackknife survey weights (50 jackknife survey weights for each year) to account for variance estimation and generalizability.

Initial weighted descriptive statistics were analyzed for all respondents. Chi-square tests were used to compare the characteristics of the study population to chronic conditions. Second, we summarized the weighted temporal prevalence of chronic diseases and digital health use in graphs. Separate weighted logistic regression models were then conducted to test the unadjusted association of the study variables and each digital health use variable. Third, multivariate logistic regression models were created to explore the adjusted association between the digital health use variables and all covariates.

Additionally, we examined the interactions between age and the chronic condition categories, race/ethnicity and income, and race/ethnicity and education and presented the adjusted predicted probabilities in plots. Lastly, we conducted a similar unadjusted and adjusted multivariate logistic regression analysis to examine the relationship between digital health use and individual chronic conditions.

Multicollinearity was also examined using the variance inflation factor, and there was no collinearity among the independent variables. Hosmer-Lemeshow goodness-of-fit test was conducted; models with insignificant chi-square test output suggest a good fit. We provided the unadjusted odds ratio (OR), adjusted odds ratio (aOR), and corresponding 95% CI. A 2-sided significance level of α<.05 for statistical significance was applied. Missing or unknown observations were dropped for all analyses except for race/ethnicity. Analyses were performed with Stata statistical software (version 17 SE; StataCorp).

## Results

### Population Characteristics

Of the 8573 women who participated in the pooled survey, the weighted prevalence and samples of those who answered “yes” to using a tablet or smartphone for various activities were as follows: achieve or track health-related goals, 50.3% (95% CI 48.4%-52.2%; 3279/7122); make a health decision, 43.6% (95% CI 41.9%-45.3%; 2998/7101); and discuss with their provider, 40% (95% CI 38.2%-41.8%; 2834/7099). In the preceding 12 months, 33% (95% CI 30.9%-35.2%; 1395/4826) of women reported using an electronic wearable device, 18.7% (95% CI 17.3%-20.2%; 1532/7653) shared health information using an electronic monitoring device, and 35.2% (95% CI 33.2%-37.3%; 2262/6349) sent or received an SMS text message with a health professional.

As seen in Table 1, higher shares of the women in this study had health insurance (8035/8460; 92.7%), were non-Hispanic White (4871/8573; 60.6%), were aged 50-64 years (2679/8449; 29.1%), earned above US $75,000 (2937/8528; 37.5%), had never smoked (5626/8499; 67.6%), and performed <150 minutes of moderate physical activity per week (6775/8419; 69.3%). Of the 8564 women who responded to the chronic condition variable, the sample and weighted prevalence of having 0, 1, and multiple chronic conditions were 2718 (37.4%), 2776 (33.4%), and 3070 (29.3%), respectively. However, the overall weighted pooled prevalence of having any chronic diseases between 2017 and 2020 was 52.2% (95% CI 0.50%-0.53%; 4756/8564; data not shown). Furthermore, those who reported having multiple chronic conditions were predominately in 2 age groups: 50-64 years (1033/3070; 10%) and >65 years (1470/3070; 10.3%). They were also predominately non-Hispanic White (1716/3070; 18.2%), had a regular provider (2463/3070; 23.7%), and performed <150 minutes of moderate physical activity (2286/3070; 21.8%). In addition, they mostly earned <US $20,000 (902/3070; 7.9%) and had a high school degree or below (1054/3070; 11.5%).

**Table 1:** Characteristics of the total population and by chronic conditions in the pooled sample.

Variable			Total, n (weighted %)		Number of chronic conditions^a^ (n=8564), n (weighted %)						*P* value	
					0		1		≥2			
Women			8573 (100)		2718 (37.4)		2776 (33.4)		3070 (29.3)			
**Age (years; n=8449)**												<.001
	18-34	1171 (23.7)		649 (12.5)		385 (8.4)		137 (2.8)				
	35-49	1724 (26.3)		801 (12.2)		538 (7.9)		385 (6.2)				
	50-64	2679 (29.1)		761 (9.1)		885 (10.1)		1033 (10)				
	>65	2875 (20.9)		475 (3.7)		926 (6.9)		1470 (10.3)				
**Marital status (n=8488)**												.001
	Not married	4409 (46.4)		1165 (16.2)		1404 (15.4)		1835 (14.9)				
	Married	4079 (53.6)		1522 (21.2)		1353 (18)		1204 (14.3)				
**Income (US $; n=8528)**												<.001
	<20,000	1749 (18.6)		346 (4.9)		497 (5.6)		902 (7.9)				
	20,000-34,999	1230 (12.8)		322 (4.4)		391 (3.82)		515 (4.6)				
	35,000-49,999	1145 (14)		315 (3.9)		377 (5.5)		451 (4.5)				
	50,000-74,999	1467 (17.2)		471 (6.2)		509 (6.1)		487 (4.9)				
	>75,000	2937 (37.5)		1246 (17.8)		990 (12.4)		700 (7.3)				
**Race/ethnicity (n=8573)**												<.001
	Asian/others	613 (7.0)		245 (3.5)		157 (2.3)		181 (1.3)				
	Hispanic	1201 (15)		510 (7.5)		336 (4.2)		355 (3.2)				
	Non-Hispanic African American or Black	1275 (11.8)		334 (3.7)		420 (3.9)		520 (4.2)				
	Non-Hispanic White	4871 (60.6)		1510 (21.3)		1641 (21.1)		1716 (18.2)				
	Missing	613 (5.6)		119 (1.4)		192 (1.9)		298 (2.3)				
**Education (n=8498)**												<.001
	High school or below	2230 (29.8)		523 (8.9)		651 (9.4)		1054 (11.5)				
	Some college degree	2471 (37.9)		701 (14)		783 (12.6)		987 (11.3)				
	College degree or above	3797 (32.3)		1475 (14.5)		1318 (11.4)		1002 (6.5)				
**Insurance (n=8460)**												.08
	No	425 (7.25)		185 (3.2)		134 (2.3)		106 (1.7)				
	Yes	8035 (92.7)		2499 (34.2)		2609 (31)		3026 (29.2)				
**Self-reported health status (n=8484)**												<.001
	Fair/poor	1428 (15.8)		141 (2.1)		331 (4.3)		956 (9.4)				
	Good	3044 (35.3)		728 (10.5)		1068 (12.8)		1248 (12.1)				
	Excellent	4012 (48.9)		1810 (24.7)		1354 (16.4)		846 (7.8)				
**Regular provider (n=8437)**												<.001
	No	2399 (32)		1087 (16.3)		766 (10.2)		543 (5.5)				
	Yes	6038 (68)		1592 (21)		1978 (23.2)		2463 (23.7)				
**Physical activity (n=8375)**												<.001
	<150 minutes per week	5961 (69.3)		1754 (24.1)		1915 (23.4)		2286 (21.8)				
	>150 minutes per week	2414 (30.7)		917 (13.5)		798 (9.9)		698 (7.3)				
**Smoking status (n=8499)**												<.001
	Never	5626 (67.6)		2020 (28.6)		1865 (22.4)		1736 (16.6)				
	Former	1906 (20.3)		463 (5.6)		583 (6.7)		860 (7.9)				
	Current	967 (12.1)		214 (3.2)		309 (4.2)		444 (4.7)				
**Diabetes (n=8419)**												<.001
	No	6775 (83.7)		2654 (37.2)		2518 (30.8)		1603 (15.7)				
	Yes	1644 (16.3)		0 (0)		213 (2.63)		1431 (13.6)				
**High blood pressure (n=8423)**												<.001
	No	4847 (66.3)		2634 (37)		1647 (23)		566 (6.3)				
	Yes	3576 (33.7)		0 (0)		1090 (10.5)		2486 (23.2)				
**Heart condition (n=8449)**												<.001
	No	7774 (93.2)		2648 (37)		2693 (32.8)		2433 (23.3)				
	Yes	675 (6.79)		0 (0)		53 (67.5)		622 (6.1)				
**Lung disease (n=8450)**												<.001
	No	7200 (86.1)		2647 (37)		2485 (29.9)		2068 (19.1)				
	Yes	1250 (13.9)		0(0)		264 (3.6)		986 (10.3)				
**Depression or anxiety (n=8436)**												<.001
	No	6129 (70.7)		2639 (36.9)		1924 (20.3)		1566 (13.4)				
	Yes	2307 (29.3)		0 (0)		822 (13.3)		1485 (16.1)				
**Cancer (n=8531)**												<.001
	No	7182 (89.8)		2709 (37.4)		2432 (30.3)		2042 (22.1)				
	Yes	1349 (10.2)		0 (0)		334 (3.1)		1015 (7.1)				
**Arthritis^b^ (n=3644)**												—^c^
	No	2384 (73.4)		—		—		—		—		
	Yes	1260 (26.6)		—		—		—		—		

^a^Total diabetes, high blood pressure, heart condition, lung disease, depression/anxiety, and cancer.

^b^2017-2018 and not totaled in the chronic condition category.

^c^Not available.

### Prevalence of Chronic Conditions and Digital Health Use Across Years

**Figure 1:**
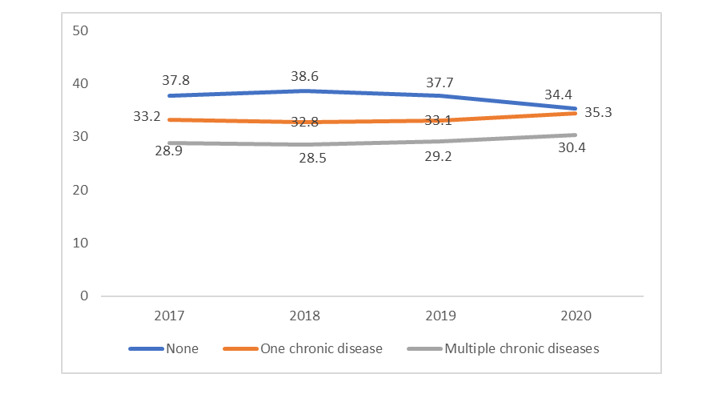
Weighted prevalence (%) of chronic disease among adult US women from 2017-2020. Chronic disease includes total diabetes, high blood pressure, heart condition, lung disease, depression or anxiety, and cancer.

Figure 1 shows the weighted prevalence of chronic conditions. Between 2017 and 2020, women without any chronic diseases declined from 37.8% (569/1784) to 34.4% (601/2047). In the same period, there was an increase in the proportion of women reporting 1 and multiple chronic conditions, from 33.2% (576/1784) to 35.3% (671/2047) and from 28.9% (639/1784) to 30.4% (775/2047), respectively. Table 2 reports the weighted prevalence of digital health use among the participants who answered “yes.” Between 2017 and 2020, there was an increase in the proportion of women who used a tablet or smartphone to achieve a health-related goal (from 596/1479; 44.4% to 866/1760; 53.8%) and discuss with their provider (from 492/1469; 33.9% to 779/1758; 44.2%). In the past 12 months, the use of electronic wearable devices increased from 30% (739/2794) to 35.9% (656/2032) between 2019 and 2020. However, there was a sharp decline from 17.5% (302/1578) in 2017 to 15.4% (309/1854) in 2020 among those who shared their health information with a health professional. 

**Table 2:** Weighted prevalence of digital health use for health promotion among adult US women: 2017-2020.

Digital health use	Weighted prevalence (%; 95% CI)			
	2017	2018	2019	2020
Used tablet to achieve goals	44.4 (40.8-48.1)	50.6 (46.5-54.7)	52.3 (51.4-55.9)	53.8 (49.7-57.9)
Used tablet to make decision	37.7 (34.3-41.3)	45.6 (41.9-49.4)	46.4 (43.3-49.6)	44.5 (41.2-47.8)
Used wearable device^a,b^	—^c^	—	30 (27.7-32.4)	35.9 (32.4-39.6)
Used tablet to discuss with provider	33.9 (30.3-37.6)	39.4 (35.3-43.6)	42.2 (39.5-45.1)	44.2 (40.2-48.3)
Shared information with provider^b^	17.5 (14.6-20.8)	20.5 (17.8-23.4)	21.8 (18.9-25)	15.4 (12.6-18.7)
Communicated via texting with provider^b,d^	33.2 (30-36.5)	33 (29.2-37.1)	39.5 (36.1-43.1)	—

^a^Only surveyed from 2019-2020.

^b^In the past 12 months.

^c^Not available.

^d^Only surveyed from 2017-2019.

### Unadjusted Odds of Digital Health Use Among Women With Chronic Conditions

In the unadjusted model (Multimedia Appendix 2), women with multiple chronic conditions were significantly less likely to use a tablet or smartphone to achieve a health goal (OR 0.74, 95% CI 0.63-0.88; *P*=.001) and use an electronic wearable device (OR 0.62, 95% CI 0.49-0.80; *P*<.001) than those with none. However, they had a higher likelihood of using their tablet or smartphone to discuss with their provider (OR 1.28, 95% CI 1.06-1.56; *P*=.01) and share health information (OR 2.02, 95% CI 1.63-2.51; *P*<.001) than women without any chronic conditions. There was a strong association between age and digital health use, and the odds were higher for women aged 18-34, 35-49, and 50-64 years (all with *P*<.001) than those aged ≥65 years. This relationship was not found when sharing their health information with a provider.

### Adjusted Odds of Digital Health Use Among Women With Chronic Conditions

After adjusting for the covariates (Multimedia Appendix 3), women with multiple chronic conditions had higher odds of using their tablet or smartphone to achieve a health-related goal (aOR 1.43, 95% CI 1.16-1.77; *P*=.001) and discuss with their provider (aOR 1.55 95% CI 1.20-2.00; *P*=.001) than those without any chronic conditions. Correspondingly, in the past 12 months, the odds of using an electronic wearable device (aOR 1.40, 95% CI 1.00-1.96; *P*=.04), sharing health information (aOR 1.91, 95% CI 1.46-2.51; *P*<.001), and communicating via SMS text messaging with a provider (aOR 1.31, 95% CI 1.02-1.68; *P*=.03) were significantly higher than women without a chronic condition. Similar to the unadjusted model, age remained a significant predictor of digital health use (*P*<.001). Our analysis also revealed that non-Hispanic Black women had significantly higher odds of digital health use than their non-Hispanic White counterparts (achieve a health related goal: *P*=.004; make a decision: *P*<.001; use a wearable device: *P*=.89; discuss with a provider: *P*<.001; share health information: *P*=.007; and communicate via SMS text messaging with a provider: *P*=.91).

Results from the subanalysis of the individual chronic condition and digital health use controlling for all covariates and the adjusted interaction models between age and chronic conditions, income and race/ethnicity, and education and race/ethnicity are shown in Multimedia Appendices 4-8.

## Discussion

### Principal Findings

This study used the latest data from the HINTS to examine the association between chronic conditions and the use of digital health tools for health promotion activities among adult US women. Our study revealed several key findings. First, we found an increasing trend of chronic condition prevalence between 2017 and 2020 from 33.2% to 35.3% and from 28.9% to 30.4% for 1 and multiple chronic conditions, respectively. We also found that slightly more than half (52.2%) of women in the United States live with a chronic disease. Following that same pattern, the overall digital health use among women has increased over time, yet interestingly, sharing information with providers has decreased. Women with 1 or multiple chronic diseases in this study had up to a 2-fold increase in using all of the digital health measures analyzed in this study compared to those without any chronic conditions. This result demonstrates that digital health technologies can provide a unique opportunity to combat chronic condition–related morbidity and mortality among women.

Overall, our results suggest that women with chronic conditions were more likely to report using digital health tools than those without these conditions. These findings are similar to previous studies showing increased digital health use, especially among those with chronic diseases [19,23-25]. A difference in digital health use was also noted based on the type of condition reported. Across the individual kinds of chronic diseases, all but cancer were strongly associated with various digital health activities. Although similar to previous studies, our results differ from 1 study showing a positive association between cancer diagnosis and digital health tools [23]. Although we may not fully understand this deviation, it is plausible that including all adult women in our model versus older women alone accounted for the difference [23]. Findings from this study suggest that although women generally accept digital health tools for numerous health activities, such as health information seeking, monitoring health conditions, patient-provider communication, or treatment, these tools are not adequately harnessed to address the growing trend of chronic diseases. In addition, since data from digital health tools can be linked to medical health records or other patient portals, our results underscore the importance of promoting its use for the continuity of medical care for chronic conditions.

Our study also explored how sociodemographic and health-related factors influence digital health use across subpopulations. Overall, younger women with higher education and income were more likely to report digital health use than older women (aged >65 years). This relationship persisted regardless of the presence of chronic conditions. Among racial/ethnic groups, non-Hispanic Black women were more likely to embrace digital health than their White counterparts. This finding should be interpreted with caution because the adjusted probability of being non-Hispanic Black, earning a higher income, and having a higher education was higher than other groups in our sample. We also found disparities in digital health use among those with more education (some college or a college degree) and incomes (>US $75,000) compared to women with lower education (high school or below) and income, respectively. These findings are similar to previous research that reports that women more likely to use digital health tools, mobile health apps, the internet, or electronic patient records are those who are younger and non-Hispanic Black and have higher education and income levels [18,19,26-28]. Age and educational differences reflect the ease, skills, and confidence in using complex digital health tools, leading to a more health-conscious and literate subpopulation that can manage their health [25,27]. This finding can guide interventions to increase the use of digital health technology to focus on digital health literacy.

Other health-related factors that increase the likelihood of digital health use were being insured, having a regular provider, being more physically active (>150 minutes/week), and being a former smoker. These findings are consistent with research on digital health use based on certain health-related factors [18,29,30]. For example, Shan and colleagues [18] found that having a regular provider was associated with at least one type of user activity. Access to a regular source of care most likely increases the likelihood of exposure to health resources and information to improve health literacy. Research supports that mobile health users are more likely to report intentions to improve diet, exercise, and lose weight, further adding to the need to increase women’s access and use of digital health tools [18,27].

Overall, our study shows that despite the increase in digital health technology, certain subpopulations experience a digital disconnect leading to health inequities. For instance, older adults are an at-risk cohort with higher disease rates and more health care needs, as well as the fastest growing group, yet they are largely disconnected from the digital world [19,23]. Low-income minority groups experience barriers due to the limited availability and affordability of mobile services and internet limitations [24]. In this current age of technology, digital inclusion and literacy have been deemed “super social determinants of health” as they address all other health determinants [11,25]. For example, access to employment, housing, or medical services apps are sometimes exclusively web-based; therefore, the inability to access these apps due to literacy level or access to a smartphone or internet shapes behaviors and health outcomes [25,31]. Thus, our results are a step in the right direction in advancing women’s health in line with the Healthy People 2030 objective [12].

### Limitations

We have several limitations in our study. HINTS is a cross-sectional survey of a nationally representative cohort of individuals; therefore, we cannot infer causality, and the directions of the associations in the study cannot be indicated. A second limitation is that there might be additional confounders that potentially influence the results of our analysis, such as geographic variations, individual motivation, digital literacy, privacy and security concerns, and health consciousness, that affect the use of digital health tools. Another limitation is that only noncommunicable chronic conditions were analyzed in this study. Ideally, we would have preferred to investigate whether digital health use varies by communicable (eg, COVID-19) and noncommunicable chronic disease status. Unfortunately, the HINTS data set did not directly ask questions related to the COVID-19 pandemic’s impact on digital health use. Although we acknowledge that the impact of the pandemic on digital health uptake may have influenced our results, we agree that the effect would be minimal considering that we analyzed multiyear data from 2017 to 2020.

Moreover, COVID-19 only minimally affected the HINTS 5, Cycle 4 (2020) data collection (see the HINTS methodological report for more details) [32]. Nonetheless, this area should be of interest for future research considering that the pandemic exerted medical, economic, and social pressures on women. Lastly, recall bias and misrepresentation by respondents are likely, considering that the survey is self-administered. Despite these limitations, our study significantly adds to the literature and, to the best of our knowledge, is the first to comprehensively assess digital health use among women with chronic conditions.

### Conclusions

As the prevalence of chronic conditions increases, especially multiple comorbidities, interventions that facilitate health promotion resulting in timely and better self-management are warranted. Despite these benefits, our study shows how women from certain subpopulations—older, low income, and uninsured—are more likely not to use digital health promotion activities. To mitigate these use disparities, Adedinsewo and colleagues [11] lay a clear blueprint on how artificial intelligence and digital tools can be harnessed across the life span to improve women’s health. These findings have useful public health implications given that chronic conditions, including cardiovascular diseases, are the leading causes of death in the United States. Therefore, these findings highlight the opportunity for researchers, policy makers, and health systems, including insurers, to prescribe successfully validated digital health apps to increase digital health use and empower women to become actively engaged in their care.

## Appendix

Multimedia Appendix 1 Outcome and key independent measures.

Multimedia Appendix 2 Unadjusted odds of digital health use for health promotion among US women: 2017-2020.

Multimedia Appendix 3 Multivariate logistic regression models of digital health use for health promotion among US women: 2017-2020.

Multimedia Appendix 4 Unadjusted logistic regression models of digital health use and the individual chronic conditions: 2017-2020.

Multimedia Appendix 5 Multivariate logistic regression models of digital health use and the individual chronic conditions: 2017-2020.

Multimedia Appendix 6 Adjusted interactions between age and chronic conditions.

Multimedia Appendix 7 Adjusted interactions between income and race/ethnicity.

Multimedia Appendix 8 Adjusted interactions between education and race/ethnicity.

## Abbreviations

aOR: adjusted odds ratio
HINTS: Health Information Trends Survey
OR: odds ratio
