# Supplementary material for: The Use of Digital Health Tools for Health Promotion Among Women With and Without Chronic Diseases: Insights From the 2017-2020 Health Information National Trends Survey
Source: JMIR Mhealth Uhealth. 2022 Aug 19;10(8):e39520. doi: 10.2196/39520 (PMC9440408; doi:10.2196/39520)
Supplement: Multimedia Appendix 1 [file mhealth_v10i8e39520_app1.docx]

The Use of Digital Health Tools for Health Promotion Among Women with and Without Chronic Diseases: Insights from the 2017-2020 Health Information National Trends Survey

Multimedia Appendix 1: Outcome and key independent measures

| Outcome measures | Variable name | Survey Question | Categorization | Year |
| --- | --- | --- | --- | --- |
| Used smartphone/tablet to track health progress | tablet_achievegoal | “Has your tablet or smartphone helped you track progress on a health-related goal such as quitting smoking, losing weight, or increasing physical activity? | Binary (Yes/no) | 2017-2020 |
| Used smartphone/tablet to make health decision | tablet_makedecision | “Has your tablet or smartphone helped you make a decision about how to treat an illness or condition?” | Binary (Yes/no) | 2017-2020 |
| Used wearable to monitor health | wearabledevtrackhealth | “In the last 12 months, have you used an electronic wearable device to monitor or track your health or activity? For example, a Fitbit, Apple Watch, or Garmin Vivofit.” | Binary (Yes/no) | 2019-2020 |
| When interacting with a clinician, smartphone or tablet helped discussion | tablet_discussionshcp | “Has your tablet or smartphone helped you in discussions with your health care provider?” | Binary (Yes/no) | 2017-2020 |
| When interacting with a clinician, shared information from smartphone or wearable | sharedhealthdeviceinfo | “Have you shared health information from either an electronic monitoring device or smartphone with a health professional within the last 12 months?” | Binary (Yes/no) | 2017-2020 |
| Sent/received text with a clinician | textfromdoctor | “Have you sent or received a text message from a doctor or other health care professional within the last 12 months?” | Binary (Yes/no) | 2017-2019 |
| Key Independent Variables: Chronic Condition | | | | |
| Diabetes or high blood sugar | medconditions_diabetes | “Has a doctor or other health professional ever told you that you had any of the following medical conditions: Diabetes or high blood sugar?” | Binary (yes/no) | 2017-2020 |
| High blood pressure or hypertension | medconditions_highbp | “Has a doctor or other health professional ever told you that you had any of the following medical conditions: High blood pressure or hypertension?” | Binary (yes/no) | 2017-2020 |
| Heart condition | medconditions_heartcondition | “Has a doctor or other health professional ever told you that you had any of the following medical conditions: A heart condition such as heart attack, angina or congestive heart failure?” | Binary (yes/no) | 2017-2020 |
| Lung disease | medconditions_lungdisease | “Has a doctor or other health professional ever told you that you had any of the following medical conditions: Chronic lung disease, asthma, emphysema, or chronic bronchitis?” | Binary (yes/no) | 2017-2020 |
| Arthritis | medconditions_arthritis | “Has a doctor or other health professional ever told you that you had any of the following medical conditions: Arthritis or rheumatism?” | Binary (yes/no) | 2017-2018 |
| Depression/anxiety | medconditions_depression | “Has a doctor or other health professional ever told you that you had any of the following medical conditions: Depression or anxiety disorder” | Binary (yes/no) | 2017-2020 |
| Cancer | everhadcancer | “Have you ever been diagnosed of having cancer?” | Binary (yes/no) | 2017-2020 |
