# Supplementary material for: The Use of Digital Health Tools for Health Promotion Among Women With and Without Chronic Diseases: Insights From the 2017-2020 Health Information National Trends Survey
Source: JMIR Mhealth Uhealth. 2022 Aug 19;10(8):e39520. doi: 10.2196/39520 (PMC9440408; doi:10.2196/39520)
Supplement: Multimedia Appendix 2 [file mhealth_v10i8e39520_app2.docx]

The Use of Digital Health Tools for Health Promotion Among Women with and Without Chronic Diseases: Insights from the 2017-2020 Health Information National Trends Survey

Multimedia Appendix 2: Unadjusted odds of digital health use for health promotion among U.S. women 2017-2020

|  | Tablet to achieve goals | | Tablet to make decision | | Use wearable device ^a,b^ | | Tablet to discuss with provider | | Share health information^b^ | | Communicate via text with provider ^b,c^ | |
| --- | --- | --- | --- | --- | --- | --- | --- | --- | --- | --- | --- | --- |
|  | OR ^d^ (95% CI ^e^) | *P* value | OR (95% CI) | *P* value | OR (95% CI) | *P* value | OR (95% CI) | *P* value | OR (95% CI) | *P* value | OR (95% CI) | *P* value |
| Chronic condition ^f^ |  |  |  |  |  |  |  |  |  |  |  |  |
| 0 | Ref ^g^ |  | Ref |  | Ref |  | Ref |  | Ref |  | Ref |  |
| 1 chronic condition | 0.95 (0.79-1.14) | .59 | 1.11 (0.99-1.42) | .06 | 0.98 (0.76-1.26) | .88 | 1.27 (1.04-1.57) | .01 | 1.47 (1.15-1.87) | .002 | 1.03 (0.84-1.26) | .71 |
| ≥2 chronic conditions | 0.74 (0.63-0.88) | .001 | 0.91 (0.76-1.09) | .33 | 0.62 (0.49-0.80) | <.001 | 1.28 (1.06-1.56) | .01 | 2.02 (1.63-2.51) | <.001 | 0.95 (0.78-1.17) | .66 |
| Age |  |  |  |  |  |  |  |  |  |  |  |  |
| 18-34 | 5.64 (4.42-7.21) | <.001 | 2.22 (1.74-2.83) | <.001 | 5.24 (3.86-7.12) | <.001 | 1.92 (1.53-2.40) | <.001 | 0.84 (0.62-1.13) | .24 | 1.92 (1.45-2.54) | <.001 |
| 35-49 | 3.82 (3.12-4.67) | <.001 | 2.50 (2.08-3.01) | <.001 | 2.92 (2.32-3.69) | <.001 | 2.20 (1.81-2.68) | <.001 | 0.91 (0.73-1.15) | .45 | 2.52 (2.04-3.11) | <.001 |
| 50-64 | 2.35 (1.93-2.85) | <.001 | 1.88 (1.54-2.28) | <.001 | 2.20 (1.70-2.84) | <.001 | 1.76 (1.49-2.09) | <.001 | 1.03 (0.84-1.25) | .76 | 1.96 (1.62-2.37) | <.001 |
| >65 | Ref |  | Ref |  | Ref |  | Ref |  | Ref |  | Ref |  |
| Marital status |  |  |  |  |  |  |  |  |  |  |  |  |
| Married | 1.09 (0.93-1.27) | .26 | 1.02 (0.88-1.20) | .71 | 1.10 (0.89-1.37) | .36 | 1.14 (0.98-1.33) | .07 | 1.16 (0.95-1.40) | .13 | 1.47 (1.23-1.76) | <.001 |
| Not married | Ref |  | Ref |  | Ref |  | Ref |  | Ref |  | Ref |  |
| Income |  |  |  |  |  |  |  |  |  |  |  |  |
| <$20k | 0.47 (0.366-0.60) | <.001 | 0.81 (0.62-1.05) | .12 | 0.30 (0.21-0.43) | <.001 | 0.72 (0.55-0.94) | .01 | 0.84 (0.63-1.13) | .27 | 0.45 (0.32-0.63) | <.001 |
| $20k-$34,999 | 0.48 (0.37-0.62) | <.001 | 1.00 (0.78-1.30) | .94 | 0.27 (0.19-0.38) | <.001 | 0.69 (0.53-0.90) | .008 | 0.93 (0.67-1.28) | .66 | 0.40 (0.30-0.52) | <.001 |
| $35k -$49,999 | 0.61 (0.47-0.79) | <.001 | 1.11 (0.86-1.44) | .39 | 0.60 (0.40-0.90) | .01 | 0.78 (0.61-0.99) | .04 | 0.77 (0.58-1.02) | .07 | 0.65 (0.50-0.85) | .002 |
| $50k - $74,999 | 0.66 (0.54-0.81) | <.001 | 1.03 (0.83-1.27) | .77 | 0.49 (0.37-0.66) | <.001 | 0.86 (0.70-1.05) | .14 | 1.18 (0.93-1.51) | .16 | 0.75 (0.59-0.96) | .02 |
| >$75k | Ref |  | Ref |  | Ref |  | Ref |  | Ref |  | Ref |  |
| Race |  |  |  |  |  |  |  |  |  |  |  |  |
| NH ^h^ Blacks | 1.11 (0.91-1.36) | .26 | 1.68 (1.36-2.07) | <.001 | 0.69 (0.48-0.98) | .04 | 1.30 (1.04-1.62) | .01 | 1.29 (1.00-1.66) | .04 | 0.85 (0.65-1.11) | .23 |
| Hispanics | 1.08 (0.87-1.34) | .46 | 1.16 (0.93-1.44) | .16 | 0.73 (0.52-1.01) | .06 | 0.76 (0.62-0.94) | .01 | 0.85 (0.65-1.12) | .26 | 0.78 (0.61-1.00) | .05 |
| Asians/others | 1.44 (1.06-1.96) | .01 | 1.31 (0.96-1.80) | .08 | 1.04 (0.67-1.61) | .85 | 1.10 (0.83-1.46) | .48 | 0.76 (0.49-1.15) | .20 | 1.00 (0.71-1.41) | .98 |
| Missing | 0.41 (0.29-0.59) | <.001 | 0.68 (0.48-0.97) | .03 | 0.42 (0.27-0.65) | <.001 | 0.59 (0.40-0.86) | .006 | 0.79 (0.52-1.19) | .27 | 0.43 (0.30-0.61) | <.001 |
| NH white | Ref |  | Ref |  | Ref |  | Ref |  | Ref |  | Ref |  |
| Education |  |  |  |  |  |  |  |  |  |  |  |  |
| College degree or more | 2.36 (1.95-2.85) | <.001 | 1.22 (1.02-1.47) | .02 | 3.68 (2.81-4.81) | <.001 | 1.77 (1.46-2.15) | <.001 | 1.34 (1.07-1.67) | .009 | 2.25 (1.79-2.84) | <.001 |
| Some college | 1.47 (1.18-1.84) | .001 | 1.15 (0.94-1.42) | .16 | 2.21 (1.57-3.10) | <.001 | 1.37 (1.08-1.73) | .008 | 1.33 (1.03-1.72) | .02 | 1.70 (1.31-2.20) | <.001 |
| Less than high school degree | Ref |  | Ref |  | Ref |  | Ref |  | Ref |  | Ref |  |
| Insurance |  |  |  |  |  |  |  |  |  |  |  |  |
| Yes | 1.04 (0.74-1.47) | .78 | 0.94 (0.66-1.35) | .77 | 1.59 (0.99-2.57) | .05 | 1.89 (1.29-2.76) | .001 | 1.52 (0.89-2.60) | .11 | 2.42 (1.74-3.40) | <.001 |
| No | Ref |  | Ref |  | Ref |  | Ref |  | Ref |  | Ref |  |
| Health status |  |  |  |  |  |  |  |  |  |  |  |  |
| Fair/good | 0.58 (0.46-0.73) | <.001 | 1.17 (0.95-1.44) | .13 | 0.35 (0.25-0.49) | <.001 | 0.86 (0.67-1.09) | .22 | 1.29 (1.01-1.64) | .03 | 0.66 (0.53-0.82) | <.001 |
| Good | 0.78 (0.67-0.92) | .004 | 0.95 (0.81-1.12) | .58 | 0.62 (0.50-0.78) | <.001 | 0.92 (0.76-1.11) | .42 | 1.30 (1.06-1.60) | .01 | 0.89 (0.74-1.07) | .22 |
| Excellent | Ref |  | Ref |  | Ref |  | Ref |  | Ref |  | Ref |  |
| Regular provider |  |  |  |  |  |  |  |  |  |  |  |  |
| Yes | 0.97 (0.81-1.15) | .74 | 1.18 (0.99-1.41) | .05 | 1.10 (0.89-1.37) | .35 | 1.93 (1.57-2.36) | <.001 | 2.32 (1.81-2.98) | <.001 | 1.57 (1.29-1.92) | <.001 |
| No | Ref |  | Ref |  | Ref |  | Ref |  | Ref |  | Ref |  |
| Physical activity |  |  |  |  |  |  |  |  |  |  |  |  |
| >150 mins per/wk | 1.53 (1.28-1.82) | <.001 | 1.09 (0.92-1.29) | .31 | 1.86 (1.46-2.36) | <.001 | 1.13 (0.95-1.34) | .15 | 1.01 (0.82-1.25) | .88 | 1.25 (1.04-1.50) | .01 |
| <150 mins per/wk | Ref |  | Ref |  | Ref |  | Ref |  | Ref |  | Ref |  |
| Smoking status |  |  |  |  |  |  |  |  |  |  |  |  |
| Current | 0.53 (0.42-0.67) | <.001 | 0.97 (0.77-1.22) | .80 | 0.35 (0.24-0.52) | <.001 | 0.97 (0.75-1.25) | .83 | 0.94 (0.70-1.27) | .72 | 0.76 (0.59-0.97) | .02 |
| Former | 0.93 (0.77-1.11) | .42 | 0.96 (0.80-1.16) | .72 | 0.97 (0.77-1.22) | .81 | 1.19 (0.99-1.44) | .06 | 1.07 (0.86-1.33) | .52 | 0.97 (0.80-1.17) | .78 |
| Never | Ref |  | Ref |  | Ref |  | Ref |  | Ref |  | Ref |  |

^a^ 2019-2020; ^b^ In the past 12 months; ^c^ 2017-2019; ^d^ odds ratio (OR); ^e^ Confidence Interval; ^f^ Chronic condition: total diabetes, high blood pressure, heart condition, lung disease, depression/anxiety, & cancer; ^g^ reference; ^h^ non-Hispanic.
