# Supplementary material for: The Use of Digital Health Tools for Health Promotion Among Women With and Without Chronic Diseases: Insights From the 2017-2020 Health Information National Trends Survey
Source: JMIR Mhealth Uhealth. 2022 Aug 19;10(8):e39520. doi: 10.2196/39520 (PMC9440408; doi:10.2196/39520)
Supplement: Multimedia Appendix 3 [file mhealth_v10i8e39520_app3.docx]

The Use of Digital Health Tools for Health Promotion Among Women with and Without Chronic Diseases: Insights from the 2017-2020 Health Information National Trends Survey

Multimedia Appendix 3: Multivariate logistic regression models of digital health use for health promotion among U.S. women: 2017-2020

|  | Tablet to achieve goals  N=6664 | | Tablet to make decision  N=6644 | | Use wearable device ^a,b^  N=4429 | | Tablet to discuss with provider  N=6643 | | Share health information^b^  N=7088 | | Communicate via text with provider ^b,c^  N= 5893 | |
| --- | --- | --- | --- | --- | --- | --- | --- | --- | --- | --- | --- | --- |
|  | AOR ^d^ (95% CI ^e^) | *P* Value | AOR (95% CI) | *P* Value | AOR (95% CI) | *P* Value | AOR (95% CI) | *P* Value | AOR (95% CI) | *P* Value | AOR (95% CI) | *P* Value |
| Chronic condition ^f^ |  |  |  |  |  |  |  |  |  |  |  |  |
| 0 | Ref ^g^ |  | Ref |  | Ref |  | Ref |  | Ref |  | Ref |  |
| 1 chronic condition | 1.28 (1.03-1.58) | .02 | 1.28 (1.04-1.56) | .01 | 1.42 (1.05-1.92) | .02 | 1.38 (1.08-1.76) | .009 | 1.41 (1.08-1.83) | .01 | 1.12 (0.89-1.42) | .30 |
| ≥2 chronic conditions | 1.43 (1.16-1.77) | .001 | 1.01 (0.80-1.27) | .90 | 1.40 (1.00-1.96) | .04 | 1.55 (1.20-2.00) | .001 | 1.91 (1.46-2.51) | <.001 | 1.31 (1.02-1.68) | .03 |
| Age |  |  |  |  |  |  |  |  |  |  |  |  |
| 18-34 | 7.29 (5.56-9.56) | <.001 | 2.52 (1.90-3.33) | <.001 | 5.93 (4.05-8.69) | <.001 | 3.00 (2.31-3.89) | <.001 | 1.28 (0.91-1.79) | .15 | 2.21 (1.60-3.06) | <.001 |
| 35-49 | 4.31 (3.41-5.44) | <.001 | 2.62 (2.09-3.29) | <.001 | 3.27 (2.45-4.37) | <.001 | 2.95 (2.35-3.71) | <.001 | 1.14 (0.88-1.47) | .31 | 2.68 (2.07-3.45) | <.001 |
| 50-64 | 2.42 (1.96-2.99) | <.001 | 1.85 (1.49-2.30) | <.001 | 2.17 (1.65-2.86) | <.001 | 1.98 (1.63-2.39) | <.001 | 1.09 (0.88-1.36) | .39 | 1.93 (1.55-2.40) | <.001 |
| >65 | Ref |  | Ref |  | Ref |  | Ref |  | Ref |  | Ref |  |
| Marital status |  |  |  |  |  |  |  |  |  |  |  |  |
| Married | 1.10 (0.92-1.32) | .25 | 1.06 (0.89-1.25) | .47 | 0.96 (0.74-1.24) | .79 | 1.09 (0.91-1.30) | .32 | 1.16 (0.93-1.45) | .17 | 1.18 (0.95-1.45) | .12 |
| Not married | Ref |  | Ref |  | Ref |  | Ref |  | Ref |  | Ref |  |
| Income |  |  |  |  |  |  |  |  |  |  |  |  |
| <$20k | 0.55 (0.38-0.79) | .002 | 0.82 (0.58-1.17) | .28 | 0.46 (0.29-0.73) | .001 | 0.92 (0.63-1.35) | .69 | 0.98 (0.65-1.48) | .93 | 0.77 (0.48-1.22) | .27 |
| $20k-$34,999 | 0.58 (0.42-0.79) | .001 | 1.06 (0.78-1.44) | .66 | 0.42 (0.27-0.65) | <.001 | 0.83 (0.61-1.15) | .27 | 1.01 (0.71-1.45) | .93 | 0.61 (0.43-0.85) | .005 |
| $35k -$49,999 | 0.68 (0.51-0.92) | .14 | 1.14 (0.86-1.51) | .34 | 0.79 (0.53-1.17) | .24 | 0.92 (0.70-1.20) | .54 | 0.74 (0.54-1.03) | .07 | 0.86 (0.64-1.16) | .34 |
| $50k - $74,999 | 0.78 (0.62-0.99) | .43 | 1.09 (0.87-1.38) | .42 | 0.58 (0.42-0.81) | .002 | 0.97 (0.76-1.23) | .82 | 1.18 (0.92-1.52) | .18 | 0.94 (0.71-1.23) | .67 |
| >$75k | Ref |  | Ref |  | Ref |  | Ref |  | Ref |  | Ref |  |
| Race |  |  |  |  |  |  |  |  |  |  |  |  |
| NH ^h^ Blacks | 1.44 (1.12-1.85) | .004 | 1.79 (1.41-2.28) | <.001 | 0.97 (0.64-1.46) | .89 | 1.57 (1.23-2.00) | <.001 | 1.49 (1.11-2.00) | .007 | 0.98 (0.70-1.37) | .91 |
| Hispanics | 1.09 (0.83-1.44) | .49 | 1.17 (0.90-1.54) | .22 | 0.80 (0.53-1.20) | .29 | 1.00 (0.61-1.15) | .95 | 1.15 (0.80-1.54) | .51 | 0.89 (0.66-1.21) | .49 |
| Asians/others | 1.25 (0.88-1.79) | .20 | 1.26 (0.91-1.74) | .14 | 0.81 (0.50-1.33) | .41 | 1.13 (0.82-1.54) | .43 | 0.87 (0.56-1.34) | .52 | 0.87 (0.59-1.28) | .49 |
| Missing | 0.90 (0.60-1.35) | .62 | 0.98 (0.65-1.48) | .94 | 0.95 (0.57-1.59) | .86 | 0.95 (0.63-1.43) | .81 | 1.07 (0.64-1.78) | .79 | 0.68 (0.41-1.11) | .13 |
| NH white | Ref |  | Ref |  | Ref |  | Ref |  | Ref |  | Ref |  |
| Education |  |  |  |  |  |  |  |  |  |  |  |  |
| College degree or more | 1.57 (1.22-2.04) | .001 | 1.05 (0.84-1.32) | .63 | 1.86 (1.29-2.69) | .001 | 1.42 (1.11-1.81) | .005 | 1.38 (1.03-1.85) | .03 | 1.52 (1.15-2.02) | .004 |
| Some college | 1.28 (1.00-1.65) | .04 | 1.07 (0.85-1.34) | .52 | 1.57 (1.09-2.25) | .01 | 1.20 (0.93-1.56) | .14 | 1.30 (0.97-1.75) | .07 | 1.38 (1.03-1.84) | .02 |
| Less than high school degree | Ref |  | Ref |  | Ref |  | Ref |  | Ref |  | Ref |  |
| Insurance |  |  |  |  |  |  |  |  |  |  |  |  |
| Yes | 1.01 (0.69-1.49) | .92 | 0.96 (0.65-1.42) | .85 | 1.48 (0.76-2.85) | .23 | 1.55 (1.01-2.38) | .04 | 1.16 (0.65-2.04) | .60 | 2.17 (1.45-3.25) | <.001 |
| No | Ref |  | Ref |  | Ref |  | Ref |  | Ref |  | Ref |  |
| Health status |  |  |  |  |  |  |  |  |  |  |  |  |
| Fair/good | 0.85 (0.64-1.12) | .25 | 1.29 (0.98-1.70) | .06 | 0.63 (0.42-0.95) | .03 | 0.82 (0.60-1.12) | .22 | 1.14 (0.84-1.55) | .38 | 0.84 (0.61-1.15) | .28 |
| Good | 0.92 (0.75-1.13) | .44 | 0.95 (0.78-1.15) | .61 | 0.81 (0.64-1.03) | .09 | 0.90 (0.73-1.12) | .37 | 1.22 (0.95-1.56) | .10 | 1.03 (0.82-1.28) | .78 |
| Excellent | Ref |  | Ref |  | Ref |  | Ref |  | Ref |  | Ref |  |
| Regular provider |  |  |  |  |  |  |  |  |  |  |  |  |
| Yes | 1.17 (0.94-1.45) | .14 | 1.35 (1.06-1.66) | .005 | 1.14 (0.87-1.50) | .32 | 1.96 (1.55-2.48) | <.001 | 2.03 (1.56-2.64) | <.001 | 1.49 (1.18-1.88) | .001 |
| No | Ref |  | Ref |  | Ref |  | Ref |  | Ref |  | Ref |  |
| Physical activity |  |  |  |  |  |  |  |  |  |  |  |  |
| >150 mins per/wk | 1.36 (1.12-1.65) | .002 | 1.11 (0.92-1.33) | .25 | 1.50 (1.16-1.95) | .002 | 1.08 (0.89-1.32) | .39 | 1.06 (0.84-1.33) | .61 | 1.14 (0.92-1.41) | .21 |
| <150 mins per/wk | Ref |  | Ref |  | Ref |  | Ref |  | Ref |  | Ref |  |
| Smoking status |  |  |  |  |  |  |  |  |  |  |  |  |
| Current | 0.66 (0.51-0.85) | .001 | 0.96 (0.74-1.25) | .77 | 0.46 (0.30-0.71) | .001 | 1.03 (0.77-1.37) | .83 | 0.85 (0.61-1.18) | .33 | 0.84 (0.63-1.13) | .25 |
| Former | 1.24 (1.02-1.52) | .03 | 1.07 (0.87-1.31) | .51 | 1.25 (0.91-1.72) | .15 | 1.24 (1.01-1.53) | .03 | 0.92 (0.72-1.16) | .50 | 0.95 (0.77-1.17) | .67 |
| Never | Ref |  | Ref |  | Ref |  | Ref |  | Ref |  | Ref |  |

^a^ 2019-2020; ^b^ In the past 12 months; ^c^ 2017-2019; ^d^ Adjusted odds ratio (AOR); ^e^ Confidence Interval; ^f^ Chronic disease: total diabetes, high blood pressure, heart condition, lung disease, depression/anxiety, & cancer; ^g^ reference; ^h^ non-Hispanic.
