# Supplementary material for: The Use of Digital Health Tools for Health Promotion Among Women With and Without Chronic Diseases: Insights From the 2017-2020 Health Information National Trends Survey
Source: JMIR Mhealth Uhealth. 2022 Aug 19;10(8):e39520. doi: 10.2196/39520 (PMC9440408; doi:10.2196/39520)
Supplement: Multimedia Appendix 4 [file mhealth_v10i8e39520_app4.docx]

The Use of Digital Health Tools for Health Promotion Among Women with and Without Chronic Diseases: Insights from the 2017-2020 Health Information National Trends Survey

Multimedia Appendix 4: Unadjusted logistic regression models of digital health use and the individual medical health conditions: 2017-2020

|  | Tablet to achieve goals | | Tablet to make decision | | Use wearable device ^a,b^ | | Tablet to discuss with provider | | Share health information ^b^ | | Communicate via text with provider ^b,c^ | |
| --- | --- | --- | --- | --- | --- | --- | --- | --- | --- | --- | --- | --- |
|  | OR ^d^  (95% CI ^e^) | P | OR  (95% CI) | P | OR  (95% CI) | P | OR  (95% CI) | P | OR  (95% CI) | P | OR  (95% CI) | P |
| Diabetes |  |  |  |  |  |  |  |  |  |  |  |  |
| No | Ref ^f^ |  | Ref |  | Ref |  | Ref |  | Ref |  | Ref |  |
| Yes | 0.80 (0.64-1.00) | .05 | 0.95 (0.76-1.18) | .65 | 0.51 (0.38-0.69) | <.001 | 0.99 (0.80-1.23) | .99 | 1.69 (1.37-2.07) | <.001 | 0.93 (0.74-1.15) | .51 |
| High blood pressure |  |  |  |  |  |  |  |  |  |  |  |  |
| No | Ref |  | Ref |  | Ref |  | Ref |  | Ref |  | Ref |  |
| Yes | 0.62 (0.53-0.73) | <.001 | 0.75 (0.64-873) | <.001 | 0.50 (0.41-0.61) | <.001 | 0.90 (0.76-1.06) | .21 | 1.47 (1.24 -1.74) | <.001 | 0.79 (0.67-0.94) | .009 |
| Heart condition |  |  |  |  |  |  |  |  |  |  |  |  |
| No | Ref |  | Ref |  | Ref |  | Ref |  | Ref |  | Ref |  |
| Yes | 0.61 (0.43-0.86) | .006 | 0.63 (0.44-0.90) | .01 | 0.42 (0.27-0.66) | <.001 | 0.71 (0.49-1.02) | .06 | 1.40 (1.02-1.93) | .03 | 0.64 (0.44-0.94) | .02 |
| Lung disease |  |  |  |  |  |  |  |  |  |  |  |  |
| No | Ref |  | Ref |  | Ref |  | Ref |  | Ref |  | Ref |  |
| Yes | 0.89 (0.72-1.12) | .35 | 1.12 (0.90-1.39) | .27 | 1.16 (0.84-1.60) | .35 | 1.21 (0.96-1.53) | .10 | 1.28 (1.01-1.62) | .03 | 1.03 (0.81-1.32) | .77 |
| Ever had cancer |  |  |  |  |  |  |  |  |  |  |  |  |
| No | Ref |  | Ref |  | Ref |  | Ref |  | Ref |  | Ref |  |
| Yes | 0.66 (0.53-0.83) | .001 | 0.80 (0.62-1.01) | .07 | 0.62 (0.40-0.95) | .03 | 0.89 (0.69-1.15) | .39 | 1.26 (0.99-1.62) | .05 | 0.94 (0.73-1.21) | .66 |
| Depression/anxiety |  |  |  |  |  |  |  |  |  |  |  |  |
| No | Ref |  | Ref |  | Ref |  | Ref |  | Ref |  | Ref |  |
| Yes | 1.26 (1.06-1.50) | .007 | 1.31 (1.10-1.57) | .003 | 1.20 (0.95-1.50) | .01 | 1.49 (1.24-1.80) | <.001 | 1.38 (1.14-1.68) | .001 | 1.36 (1.14-1.62) | .001 |
| Arthritis ^g^ |  |  |  |  |  |  |  |  |  |  |  |  |
| No | Ref |  | Ref |  | - | - | Ref |  | Ref |  | Ref |  |
| yes | 0.66 (0.52-0.85) | .002 | 0.87 (0.68-1.10) | .24 | - | - | 0.98 (0.75-1.28) | .90 | 1.49 (1.13-1.96) | .005 | 0.91 (0.71-1.16) | .46 |

^a^ 2019-2020; ^b^ In the prior 12 months; ^c^ 2017-2019; ^d^ Odds Ratio (OR); ^e^ Confidence Interval; ^f^ Reference; ^g^ 2017-2018.
