# Supplementary material for: The Use of Digital Health Tools for Health Promotion Among Women With and Without Chronic Diseases: Insights From the 2017-2020 Health Information National Trends Survey
Source: JMIR Mhealth Uhealth. 2022 Aug 19;10(8):e39520. doi: 10.2196/39520 (PMC9440408; doi:10.2196/39520)
Supplement: Multimedia Appendix 5 [file mhealth_v10i8e39520_app5.docx]

The Use of Digital Health Tools for Health Promotion Among Women with and Without Chronic Diseases: Insights from the 2017-2020 Health Information National Trends Survey

Multimedia Appendix 5: Multivariate logistic regression models of digital health use and the individual medical health conditions: 2017-2020

|  | Tablet to achieve goals | | Tablet to make decision | | Use wearable device ^a,b^ | | Tablet to discuss with provider | | Share health information ^b^ | | Communicate via text with provider ^b,c^ | |
| --- | --- | --- | --- | --- | --- | --- | --- | --- | --- | --- | --- | --- |
|  | AOR ^d^ (95% CI ^e^) | P | AOR (95% CI) | P | AOR (95% CI) | P | AOR (95% CI) | P | AOR (95% CI) | P | AOR  (95% CI) | P |
| Diabetes |  |  |  |  |  |  |  |  |  |  |  |  |
| No | Ref ^f^ |  | Ref |  | Ref |  | Ref |  | Ref |  | Ref |  |
| Yes | 1.32 (1.03-1.69) | .02 | 1.00 (0.79-1.28) | .94 | 0.95 (0.67-1.33) | .77 | 1.14 (0.89-1.46) | .29 | 1.59 (1.24-2.05) | <.001 | 1.17 (0.91-1.52) | .20 |
| High blood pressure |  |  |  |  |  |  |  |  |  |  |  |  |
| No | Ref |  | Ref |  | Ref |  | Ref |  | Ref |  | Ref |  |
| Yes | 1.10 (0.91-1.32) | .29 | 0.83 (0.68-1.01) | .07 | 1.06 (0.82-1.36) | .62 | 1.04 (0.86-1.26) | .65 | 1.39 (1.15-1.68) | .001 | 1.10 (0.90-1.34) | .32 |
| Heart condition |  |  |  |  |  |  |  |  |  |  |  |  |
| No | Ref |  | Ref |  | Ref |  | Ref |  | Ref |  | Ref |  |
| Yes | 1.08 (0.73-1.59) | .67 | 0.69 (0.47-1.01) | .05 | 0.86 (0.46-1.58) | .62 | 0.82 (0.55-1.24) | .36 | 1.42 (0.98-2.06) | .06 | 0.93 (0.61-1.43) | .76 |
| Lung disease |  |  |  |  |  |  |  |  |  |  |  |  |
| No | Ref |  | Ref |  | Ref |  | Ref |  | Ref |  | Ref |  |
| Yes | 1.03 (0.79-1.35) | .77 | 1.14 (0.90-1.45) | .25 | 1.50 (1.04-2.15) | .02 | 1.21 (0.93-1.57) | .14 | 1.19 (0.92-1.54) | .16 | 1.11 (0.84-1.47) | .44 |
| Ever had cancer |  |  |  |  |  |  |  |  |  |  |  |  |
| No | Ref |  | Ref |  | Ref |  | Ref |  | Ref |  | Ref |  |
| Yes | 1.09 (0.85-1.39) | .49 | 0.92 (0.70-1.20) | .54 | 0.94 (0.58-1.52) | .80 | 1.02 (0.78-1.35) | .83 | 1.08 (0.84-1.40) | .51 | 1.19 (0.89-1.58) | .23 |
| Depression/anxiety |  |  |  |  |  |  |  |  |  |  |  |  |
| No | Ref |  | Ref |  | Ref |  | Ref |  | Ref |  | Ref |  |
| Yes | 1.36 (1.10-1.67) | .003 | 1.28 (1.05-1.56) | .01 | 1.25 (0.96-1.63) | .08 | 1.41 (1.13-1.76) | .002 | 1.33 (1.07-1.66) | .009 | 1.34 (1.09-1.64) | .005 |
| Arthritis ^g^ |  |  |  |  |  |  |  |  |  |  |  |  |
| No | Ref |  | Ref |  | - | - | Ref |  | Ref |  | Ref |  |
| yes | 1.29 (0.95-1.74) | .95 | 1.10 (0.79-1.51) | .55 | - | - | 1.25 (0.99-1.69) | .13 | 1.41 (1.00-2.00) | .04 | 1.40 (1.04-1.90) | .02 |

^a^ 2019-2020; ^b^ In the prior 12 months, ^c^ 2017-2019; ^d^ Adjusted Odds Ratio (aOR); ^e^ Confidence Interval; ^f^ Reference; ^g^ 2017-2018; All models adjusted for age, marital status, income, race, education, insurance, health status, regular provider, physical activity, and smoking status
