# Supplementary material for: The Use of Digital Health Tools for Health Promotion Among Women With and Without Chronic Diseases: Insights From the 2017-2020 Health Information National Trends Survey
Source: JMIR Mhealth Uhealth. 2022 Aug 19;10(8):e39520. doi: 10.2196/39520 (PMC9440408; doi:10.2196/39520)
Supplement: Multimedia Appendix 6 [file mhealth_v10i8e39520_app6.docx]

The Use of Digital Health Tools for Health Promotion Among Women with and Without Chronic Diseases: Insights from the 2017-2020 Health Information National Trends Survey


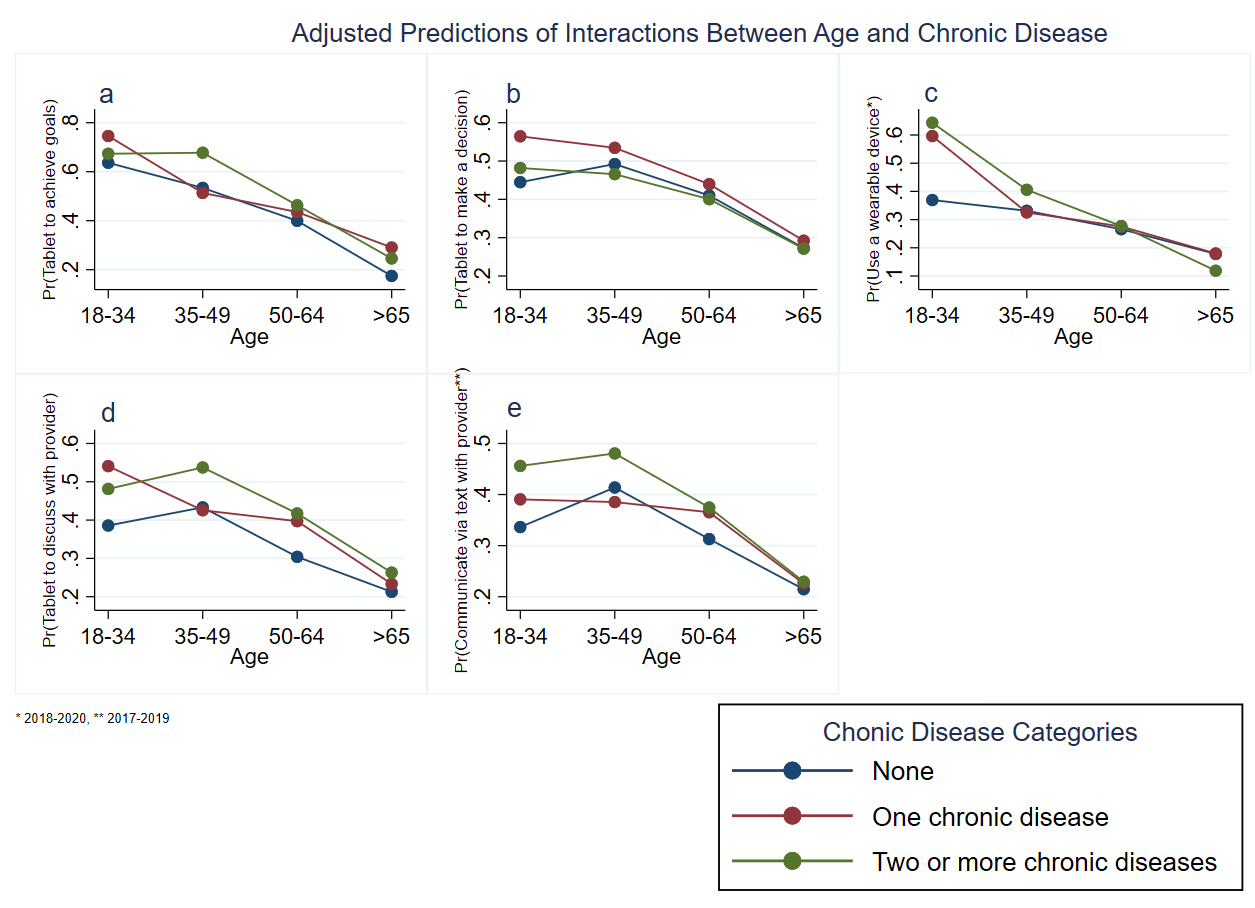


Multimedia Appendix 6: Adjusted interactions between age and chronic disease
